# A systematic review of immune-based interventions for perinatal neuroprotection: closing the gap between animal studies and human trials

**DOI:** 10.1186/s12974-023-02911-w

**Published:** 2023-10-20

**Authors:** Sharmony B. Kelly, Nhi T. Tran, Graeme R. Polglase, Rodney W. Hunt, Marcel F. Nold, Claudia A. Nold-Petry, David M. Olson, Sylvain Chemtob, Gregory A. Lodygensky, Sarah A. Robertson, Alistair J. Gunn, Robert Galinsky

**Affiliations:** 1 The Ritchie Centre, Hudson Institute of Medical Research, 27-31 Wright Street, Clayton, Melbourne, VIC 3168 Australia; 2 Department of Obstetrics and Gynaecology, Monash University, Melbourne, VIC Australia; 3 Department of Paediatrics, Monash University, Melbourne, VIC Australia; 4 Monash Newborn, Monash Children’s Hospital, Melbourne, Australia; 5 Department of Obstetrics and Gynaecology, University of Alberta, Edmonton, Canada; 6 Department of Paediatrics, CHU Sainte Justine Research Centre, University of Montreal, Quebec, Canada; 7 The University of Adelaide, Robinson Research Institute, North Adelaide, SA Australia; 8 Department of Physiology, The University of Auckland, Auckland, New Zealand

**Keywords:** Hypoxia–ischaemia, Infection/inflammation, Newborn brain, Neuroinflammation, Neuroprotection, Anti-inflammatory, Immunomodulation, Antibiotics, Corticosteroids, Interleukin-1, Tumour necrosis factor

## Abstract

**Background:**

Perinatal infection/inflammation is associated with a high risk for neurological injury and neurodevelopmental impairment after birth. Despite a growing preclinical evidence base, anti-inflammatory interventions have not been established in clinical practice, partly because of the range of potential targets. We therefore systematically reviewed preclinical studies of immunomodulation to improve neurological outcomes in the perinatal brain and assessed their therapeutic potential.

**Methods:**

We reviewed relevant studies published from January 2012 to July 2023 using PubMed, Medline (OvidSP) and EMBASE databases. Studies were assessed for risk of bias using the SYRCLE risk of bias assessment tool (PROSPERO; registration number CRD42023395690).

**Results:**

Forty preclinical publications using 12 models of perinatal neuroinflammation were identified and divided into 59 individual studies. Twenty-seven anti-inflammatory agents in 19 categories were investigated. Forty-five (76%) of 59 studies reported neuroprotection, from all 19 categories of therapeutics. Notably, 10/10 (100%) studies investigating anti-interleukin (IL)-1 therapies reported improved outcome, whereas half of the studies using corticosteroids (5/10; 50%) reported no improvement or worse outcomes with treatment. Most studies (49/59, 83%) did not control core body temperature (a known potential confounder), and 25 of 59 studies (42%) did not report the sex of subjects. Many studies did not clearly state whether they controlled for potential study bias.

**Conclusion:**

Anti-inflammatory therapies are promising candidates for treatment or even prevention of perinatal brain injury. Our analysis highlights key knowledge gaps and opportunities to improve preclinical study design that must be addressed to support clinical translation.

**Supplementary Information:**

The online version contains supplementary material available at 10.1186/s12974-023-02911-w.

## Introduction

Perinatal inflammation is highly associated with neonatal mortality and morbidity, including neurodevelopmental disorders such as vision and hearing impairments, learning difficulties, autism spectrum disorder, behavioural hyperactivity, schizophrenia and cerebral palsy (CP) [1–3]. Of particular concern, the risk of CP is increased several-fold in both preterm and term infants exposed to perinatal inflammation (odds ratio: 2.5–9.3) [4–6]. The cumulative lifetime economic cost of CP in the USA was estimated to be over USD 11.5 billion in 2003 [3]. More recent evidence indicates that the cost of disability associated with perinatal brain injury continues to rise, and that prevention of such injury would substantially reduce the socio-economic burden on affected individuals, their families and society [7].

The only commonly used treatment for targeting inflammation, namely corticosteroids (glucocorticoids), may exacerbate brain injury and increase the risk of cerebral palsy [8]. Magnesium sulphate for preterm neuroprotection, currently recommended for maternal administration when preterm labour is expected before 30 weeks of gestation, may in part act through inhibition of the NF-*κ*B inflammatory pathway [9, 10]. However, recent follow-up studies to school age suggest it does not significantly improve longer-term neurodevelopmental outcomes compared to placebo [11, 12], although these studies are relatively small due to incomplete follow-up. Conversely, both small and large animal studies suggest that therapeutic hypothermia is not neuroprotective after exposure to perinatal infection/inflammation at term [13–16]. Collectively, these data suggest that current therapeutics aimed at improving neurodevelopmental outcomes in preterm and term infants are at best partially effective, and that development of targeted anti-inflammatory treatments is an important area of unmet medical need [17, 18].

There is strong evidence that chronic inflammation related to perinatal infection and hypoxia–ischaemia can independently or synergistically cause inflammation in the fetus and neonate [19, 20]. In recent cohort studies, long-term neurodevelopmental disturbances were associated with chronic systemic inflammation and diffuse injury in the white matter tracts in both term and preterm infants [2, 6, 21–24]. As previously described, both systemic and central nervous system inflammation are strongly associated with cell death, dysmaturation and disturbed neuronal and oligodendrocyte development and reductions in brain growth [25–28]. These disturbances in white and grey matter development at the cellular level likely underpin altered brain microstructure, reduced white and grey matter volumes [29, 30] and long-term behavioural and intellectual disabilities after exposure to perinatal inflammation.

Despite this strong preclinical evidence that exposure to inflammation does trigger brain injury, and encouraging preclinical studies, no anti-inflammatory interventions have been shown to prevent clinical perinatal brain injury. In part this reflects confusion about the most appropriate drug targets and lack of clarity on the most appropriate preclinical studies to provide a foundation for safety and efficacy trials in humans. In this systemic review we aimed to evaluate the rigour of preclinical studies undertaken in the last 10 years that investigated potential immunomodulatory therapeutics to reduce perinatal inflammation-induced brain injury. A secondary aim was to determine the current knowledge gaps for clinical translation of the identified therapeutics.

## Analysis strategy

### Search method

This systematic review was conducted according to the Preferred Reporting Items for Systematic Reviews and Meta-Analysis (PRISMA) guidelines [31] (Additional file 1: Table S1 and Additional file 2: Table S2). The protocol was developed and registered with the International Prospective Register of Systematic Reviews (PROSPERO; registration number CRD42023395690).

Searches were conducted using Pubmed, Medline (OvidSP) and EMBASE databases for publications between January 2012 and July 2023. The following search terms: (preterm brain injury OR perinatal encephalopathy OR neonatal encephalopathy) AND (anti-inflammatory) were utilised. Other sources used to identify studies included relevant manuscripts and reviews. Reviews, conference abstracts, and articles written in a language other than English or for which no translation was available were excluded. Search results for both databases were collated, and duplicate articles were manually removed. Abstracts were identified and screened by an unbiased investigator (SBK) and duplicated by another investigator (NTT).

### Selection criteria

Studies were deemed eligible if they met the following criteria: (1) conducted in an in vivo model of preterm/term equivalent age; (2) intervention possesses immunomodulatory or antimicrobial effects, or exclusively impacts immune activation (Table 3); (3) clear histological (based on the assessment of tissue inflammation and injury) and/or functional outcomes are reported; and (4) comparison to a vehicle control group is made. Studies were excluded if they: (1) were conducted in vitro; (2) did not meet the age criteria (i.e. were conducted in adult/paediatric equivalent subjects); (3) tested drugs reported to have therapeutic impacts beyond immunomodulation; (4) did not report outcomes relating to neuroinflammation and related brain injury, or (5) did not include appropriate control groups. In vitro studies were excluded from this analysis due to their limited ability to capture complex interactions between systemic immune activation and brain pathophysiology.

### Data extraction

Studies were grouped by therapeutic agent and then further subdivided by species, age, type of insult to induce inflammation/injury, treatment and dosing regimen, extent of temperature monitoring, subject sex and main study outcomes (pathological/functional) and outcome (protection/no protection). The (SYstematic Review Centre for Laboratory animal Experimentation) SYRCLE risk of bias tool, described below, was used to evaluate the potential for individual study bias.

Studies were assessed according to the extent of temperature control, whether the insult and treatment were randomised, whether investigators were blinded to the intervention during histological and or functional assessments, and whether males and females were included in the analysis.

Studies were defined as being neuroprotective if there was a statistically significant improvement (*P* < 0.05) in brain histopathology and/or functional outcomes in the insult group that received treatment compared to the insult group that received vehicle/placebo.

### Risk of bias

A risk of bias assessment for the selected studies was conducted using the SYRCLE Risk of Bias (RoB) tool [32]. The SYRCLE’s RoB tool assesses the quality of animal studies (e.g. randomisation and blinding procedures in study design) to critically appraise the preclinical research methodology. The 10 RoB assessment domains were scored as either “yes” for low risk of bias, “no” for high risk of bias, or “unclear” if the experimental methods did not explicitly address the domain assessment (Table 3).

## Results

**Fig. 1:**
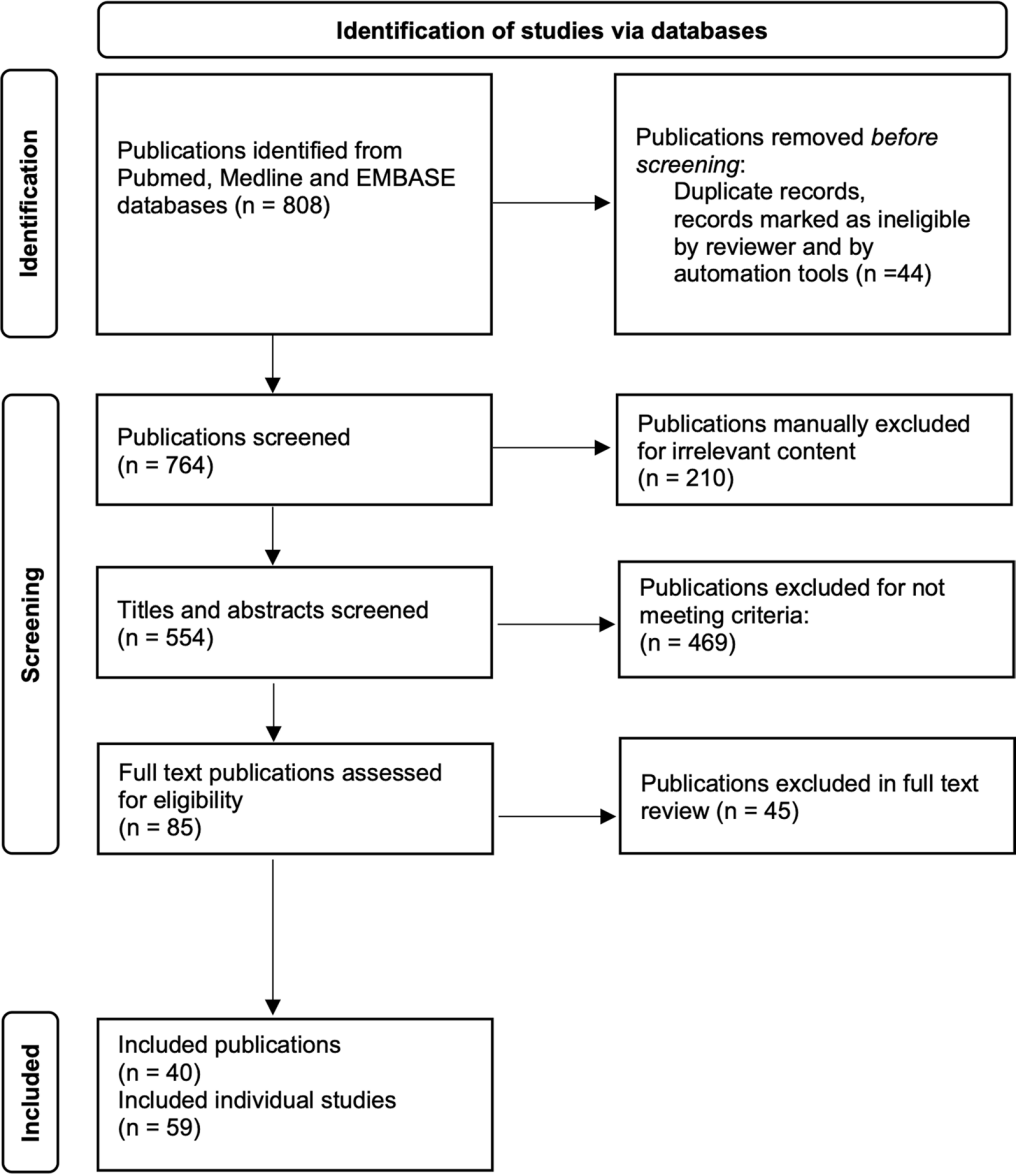
Flowchart illustrating the number of papers identified through database searching and other relevant sources, the number of full text articles screened, assessed, and excluded, and the final number of original papers surveyed. Publications that used more than one paradigm of encephalopathy or multiple treatment regimens were further subdivided if outcomes differed according to experimental paradigm or treatment regimen. After subdividing these publications there was a total of 59 individual studies. The studies are summarised in Table 1

**Fig. 2:**
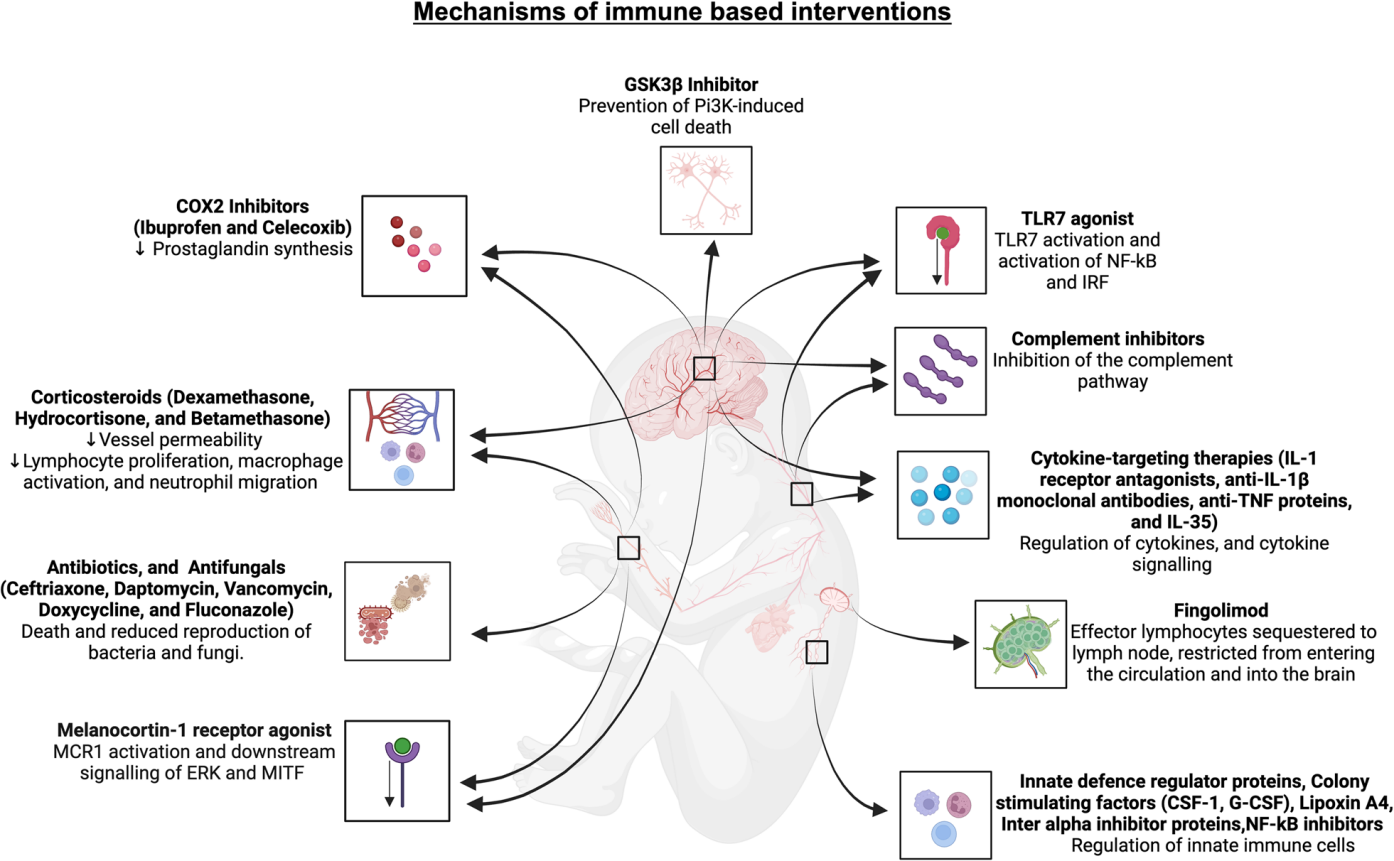
Outline of systemic and central nervous system inflammatory responses targeted by the immune-based therapeutics identified in this systematic review. Created with BioRender.com

We identified 808 relevant records. After excluding reviews, duplicates, and records for which the full text was not available, we screened a total of 764 records and excluded 724 for one or more of the following reasons: ex vivo studies, inappropriate developmental age, brain histology and functional outcomes were not examined, or the therapeutic under investigation did not explicitly affect the immune system. A total of 40 publications investigating 19 categories of therapeutic were included in this analysis (Figs. 1 and 2). Publications that used more than one model of injury or showed different outcomes based on different treatment regimens (e.g. different drug dose and timing of drug delivery) were further subdivided into individual studies. The original 40 publications were thereby subdivided into 59 individual studies, which are summarised in Table 1.

**Table 1:** Studies of immunomodulatory therapies for treatment of perinatal inflammation-induced brain injury

Reference	Species, *n*	Insult	Treatment and dose	Treatment timing	Temp (T)	Sex	Pathological outcomes	Functional outcomes	Assessment time	Protective
*Anti-fungal*										
[61]	Fetal sheep 0.8 GA	*C. albicans i.a*	Fluconazole 30 mg, i.a	2 d after insult	–	–	↑ Iba-1 staining and ↓CNPase in white matter ↑ GFAP and Iba-1 staining in hippocampus in C.alb + flu (*n* = 5–6) vs. C.alb + veh (*n* = 6)	–	3 and 5 d	No
*Corticosteroids*										
[54]	Rat pups, P5	LPS, 1 μg, i.c	Dexamethasone, 0.5 mg/kg, i.p.,	1 h before insult	–	–	↓Microglial activation ↑Myelination in dex + LPS vs. veh + LPS (ns = not reported)	↑Memory and sensory motor function in dex + LPS vs veh + LPS (ns = not reported)	1, 3, and 9 d	Yes
[54]	Rat pups, P5	LPS, 1 μg, i.c	Betamethasone, 0.5 mg/kg, i.p.,	1 h before insult	–	–	↓Microglial activation ↑Myelination in beta + LPS vs veh + LPS (ns = not reported)	↑Memory and sensory motor function in beta + LPS vs veh + LPS (ns = not reported)	1, 3, and 9 d	Yes
[63]	Rat pups, P7	HI, CAL, 8% O_2_, 2 h,	Dexamethasone, 0.5–0.1 mg/kg, i.p., daily	Days 3–1 before insult	Ambient T: 37 ℃ during HI	M	↑Cell death DEX + HI vs. veh + HI (ns = not reported)	–	1 d	No
[62]	Rat pups, P7	HI, CAL, 8% O_2_, 120–150 min	Dexamethasone 1–30 µg, i.n	2 h after insult	–	M + F	No improvement in infarct size HI + DEX (*n* = 8–14) vs. HI + veh (*n* = 8–17)	–	2 d	No
[62]	Rat pups, P7	HI, CAL, 8% O_2_, 120–150 min	Hydrocortisone, 30 µg, ICV	2 h after insult	–	M + F	↓Infarct size HI + HCZ (*n* = 10) vs. HI + veh (*n* = 11)	–	2 d	Yes
[62]	Rat pups, P7	HI, CAL, 8% O_2_, 120–150 min	Hydrocortisone, 50 µg, 100 µg, 300 µg, i.n.,	2 h after insult	–	M + F	↓Infarct size with 300 µg in ♂ HI + HCZ (*n* = 10–17) vs. HI + veh (*n* = 17)	–	2 d	Yes
[62]	Rat pups, P7	LPS i.p. + HI (CAL, 8% O_2_, 120–150 min)	Hydrocortisone, 300 µg, 1000 µg, i.n	2 h after insult	–	M + F	↓Infarct size with 300 µg HI + HCZ (*n* = 18) vs. HI + veh (*n* = 23)	–	2 d	Yes
[43]	Fetal sheep, 0.7 GA	Umbilical cord occlusion, 25 min	Maternal dexamethasone, 12 mg, i.m	15 min after insult	–	M + F	↓NeuN + cells in the hippocampus and striatum ↓Oligodendrocytes in PVWM HI + DEX (*n* = 10) vs. HI + veh (*n* = 10)	↑ Proportion of slow wave seizure-like activity	1 wk	No
[44]	Fetal sheep, 0.7 GA	Umbilical cord occlusion, 25 min	Maternal dexamethasone, 12 mg, i.m	4 h before insult	–	M + F	↑White and grey matter tissue loss dex + HI (*n* = 7) vs veh + HI (*n* = 9)	↑Seizures	1 wk	No
[64]	Newborn lambs 0.8 GA,	High tidal volume mechanical ventilation	Maternal betamethasone × 2 11.2 mg, i.m. 24 h apart	2 and 1 d before insult	Core T: 38–39 ℃	M + F	↑ Iba-1 and GFAP in white matter ↑ vascular extravasation ↑ CSF oxidative stress BM + vent (*n* = 7) vs. saline + vent (*n* = 5)	–	1 h 25 min	No
*Inter-Alpha Inhibitor Proteins*										
[65]	Rat pups, P7	HI, CAL, 8% O_2_, 90 min,	Inter-alpha inhibitor protein, 30 mg/kg, i.p	Immediately, then 1 and 2 d after insult	Ambient T: 36 ℃ during HI	M + F	↓Infarct volume, ↓cell death in the cortex and ↑myelin density in corpus callosum and internal capsule in males; HI + IAPS (*n* = 43) vs HI + veh (*n* = 39)	–	3 d	Yes
[65]	Rat pups, P7	HI, CAL, 8% O_2_, 90 min,	Inter-alpha inhibitor protein, 30 mg/kg, i.p	6 h, then 1, and 2 d after insult	Ambient T: 36 ℃ during HI	M + F	No improvement in pathological score HI + IAPS (*n* = 35) vs HI + veh (*n* = 33) groups	–	3 d	No
[66]	Rat pups, P7	HI, CAO, 8% O_2_, 120 min	Inter-alpha inhibitor protein, 30 mg/kg, i.p	1 and 25 h after insult	Core T: 36 ℃ during HI	M	–	↑Working memory in juveniles HI + IAPS (*n* = 13) vs HI + veh (*n* = 10)	81 d	Yes
[66]	Rat pups, P7	HI, CAO, 8% O_2_, 120 min	Inter-alpha inhibitor protein, 30 mg/kg, i.p	1 and 25 h after insult	Core T: 36 ℃ during HI	M	–	No improvement in working memory in adolescents HI + IAPS (*n* = 10) vs HI + veh (*n* = 12)	102 d	No
*Complement inhibitors*										
[67]	Rat pups, P10	HI, CAL, 8% O_2_, 45 min	RLS-0071, 2 doses of 10 mg/kg, 4 h apart, i.p	1 h after insult	Ambient T: 37 ℃	M + F, Ns not reported	No improvement in cortical infarct area No improvement in lesion volume or tissue oedema HI + RLS-0071 (*n* = 9) vs HI + veh (*n* = 9)	No improvement in cognition OR motor function HI + RLS-0071 (*n* = *n* = 4–7) vs HI + veh (*n* = 3–18)	Pathology: 2 d MRI: 1 and 3 d Cognition: 6 wks	No
[67]	Rat pups, P10	HI, CAL, 8% O_2_, 45 min, *n* = 9	RLS-0071, 2 doses of 10 mg/kg, 4 h apart, i.p. AND hypothermia, *n* = 9	1 h after insult	Core T: 28–30 °C	M + F, Ns not reported	↓Cortical infarct area ↓Lesion volume HI + RLS-0071 + HT (*n* = 10) vs. HI + veh + HT (*n* = 9)	↑Cognition HI + RLS-0071 + HT (*n* = 12) vs. HI + veh + HT (*n* = 20) No improvement in motor function	Pathology: 2 d MRI: 1 d Cognition: 6 wks	Yes
*TLR7 agonist*										
[41]	Fetal sheep, 0.7 GA	Umbilical cord occlusion, 25 min	GDQ, 1.8 mg/kg, ICV	1 h after insult	Not reported	M + F	↑Olig2, ↓Caspase-3 ↓GFAP ↑Neurons in caudate nucleus, dentate gyrus and thalamus UCO + GDQ (*n* = 7) vs. UCO + veh (*n* = 9)	–	3 d	Yes
[42]	Fetal sheep, 0.7 GA	Umbilical cord occlusion, 25 min,	GDQ, 1 mg/kg, ICV	1 h after insult	Not reported	M + F	↓Neurons in striatum and hippocampus UCO + GDQ (*n* = 7) vs. UCO + veh (*n* = 7)	↑Epileptiform discharges UCO + GDQ (*n* = 7) vs. UCO + veh (*n* = 7)	1 wk	No
*Antibiotics*										
[58]	Mouse pups, P4	*S. epidermidis, i.p.* + HI,	Vancomycin, 15 mg/kg, i.p	2 min after insult	Core T monitored throughout	M + F	↓ % tissue loss in cortical and deep grey matter and white matter S.epi + HI + Van (*n* = 11) vs. S.epi + HI + veh (*n* = 9)	–	9 d	Yes
[58]	Mouse pups, P4	*S. epidermidis, i.p.* + HI	Vancomycin, 15 mg/kg, i.p, + pentoxifylline 40 mg/kg, i.p	2 min after insult	Core T monitored throughout	M + F	No improvement in % tissue loss in cortical and deep grey matter or white matter S.epi + HI + Van + PTX (*n* = 9) vs. S.epi + HI + Van + veh (*n* = 9)	–	9 d	No
[68]	Rat pups, P7	HI, CAL, 8% O_2_ 60 min	Doxycycline, 10 mg/kg, i.p.,	1 h after insult	Ambient 36 ± 0.5 °C during hypoxia	M + F	↓Lesion size and ↓neuronal loss in HI + doxy (*n* = 8) vs HI + veh (*n* = 8)	–	42 d	Yes
[57]	Rat pups, P11	*S. pneumonia*, i.c.,	Ceftriaxone, 100 mg/kg, i.p. + daptomycin, 10 mg/kg/, s.c,	18 h after insult	Ambient T: 22 ± 2 °C	M + F	↓ Cortical necrosis, no difference in hippocampal apoptosis S.pne + CRO + DAP (*n* = 18) vs. S.pne + CRO (*n* = 12)	–	42 h	Yes
[57]	Rat pups, P11	*S. pneumonia*, i.c	Ceftriaxone, 100 mg/kg, i.p. + Trocade (MMP inhibitor), 2 × 75 mg/kg/d, i.p	18 h after insult	Ambient T: 22 ± 2 °C	M + F	↓Hippocampal apoptosis, no difference in cortical necrosis S.pne + CRO + TRO (*n* = 19) vs. S.pne + CRO (*n* = 12)	–	42 h	Yes
[57]	Rat pups, P11	*S. pneumonia*, i.c	Ceftriaxone, 100 mg/kg, i.p. + daptomycin, 10 mg/kg, s.c. + Trocade (MMP inhibitor), 2 × 75 mg/kg/d, i.p	18 h after insult	Ambient T: 22 ± 2 °C	M + F	↓Hippocampal apoptosis ↓Cortical necrosis ↓CSF IL-1β, TNF, IL-6, and IL-10 in the CSF S.pne + CRO + DAP + TRO (*n* = 35) vs. S.pne + CRO (*n* = 12)	–	42 h	Yes
[57]	Rat pups, P11	*S. pneumoniae*, i.c	Ceftriaxone, 100 mg/kg, i.p	18 h after insult	Ambient T: 22 ± 2 °C	M + F	–	↓Learning and memory S.pne + CRO (*n* = 14) vs. S.pne + veh (*n* = 20)	3 wks	No
[57]	Rat pups, P11	*S. pneumonia*, i.c	Ceftriaxone, 100 mg/kg, i.p. + daptomycin, 10 mg/kg, s.c. + Trocade (MMP9 inhibitor) 2 × 75 mg/kg/d, i.p	18 h after insult	Ambient T: 22 ± 2 °C	M + F	–	↑Learning and memory S.pne + CRO + DAP + TRO (*n* = 15) vs. S.pne + CRO (*n* = 14)	3 wks	Yes
*NF-κB inhibitors*										
[59]	Rat pups, P7	LPS, 0.3 mg/kg i.p. + HI 4 h later (CAL, 10%, 90 min)	Tat-NBD peptide, 1.4 mg/kg, i.n.,	10 min after insult	Ambient T: 37–38 °C during hypoxia	M + F, Ns not reported	↓% tissue loss in the cortex, hippocampus, and striatum LPS + HI + Tat-NBD (*n* = 10) vs LPS + HI + veh (*n* = 10)	–	1 wk	Yes
[59]	Rat pups, P7	LPS, 0.3 mg/kg i.p. + HI 72 h later (CAL, 10% O_2_, 90 min)	Tat-NBD peptide, 1.4 mg/kg, i.n	10 min after insult	Ambient T: 37–38 °C during hypoxia	M + F, Ns not reported	↓% tissue loss in the cortex, hippocampus, and striatum LPS + HI + Tat-NBD (*n* = 10) vs LPS + HI + veh (*n* = 10)	–	1 wk	Yes
[59]	Rat pups, P7	HI, CAL, 10% O_2_, 90 min	Tat-NBD peptide, 1.4 mg/kg, i.n	10 min after insult	Ambient T: 37–38 °C during hypoxia	M + F, Ns not reported	No improvement in % tissue loss or MMP9 activity between HI + Tat-NBD (*n* = 4–10) vs. HI + veh (*n* = 4–10)	–	1 wk	No
*Fingolimod*										
[47]	Fetal rats E17	Maternal LPS, 1 mg/kg, i.p	Maternal FTY720, 4 mg/kg, i.p	30 min after insult	–	–	↓IL-6, ↓Caspase-3 and ↓S100B + cells in white matter LPS + FTY720 (*n* = 29) vs. LPS + veh (*n* = 20)	–	6 h	Yes
[48]	Fetal rats, E17	Maternal LPS, 1 mg/kg, i.p.,	Maternal FTY720, 1 mg/kg, i.p	Immediately after insult	Ambient T: of 21–22 °C	–	↓S100β, ↓IL-6 and ↓IL-10 in the cortex LPS + FTY720 (*n* = 4) vs. LPS + veh (*n* = 4)	–	4 h	Yes
[70]	Mouse pups, P9	HI, CAL, 10% O_2_, 1 h	FTY720, 1 mg/kg, i.p	20 min after insult	Core T maintained during hypoxia	M + F	↑Tissue loss in cortex ↑Brain injury score in cortex and hippocampus ↓ MAP2 and MBP protein expression HI + FTY720 (*n* = 14) vs. HI + veh (*n* = 14)	–	1 wk	No
[37]	Rat pups, P10	Hypoxia, 5% O_2_, 15 min	FTY720, 0.3 mg/kg, i.p	45 min after HI, then daily for 11 d	–	M + F	↓TNF in the hippocampus HI + FTY720 (*n* = 13) vs. HI + veh (*n* = 16)	↓Seizures ↓Anxiety-like behaviour and ↑Memory HI + FTY720 (*n* = 12) vs. HI + veh (*n* = 16–26)	7 wks	Yes
*GSK3β inhibitor*										
[71]	Mouse pups, P9	HI, CAL, 10% O_2_, 20 min	SB216763, 10 mg/kg, i.p	14 h and immediately before, then immediately and 3 h after insult	Ambient T: 36 °C during hypoxia	–	↓Caspase-3 in the cortex and hippocampus at 6 h ↓TNFα, IL-6 mRNA and STAT3 in cortex and hippocampus at 6 h ↓% tissue loss at 7 d in SB216763 + HI (*n* = 8) vs veh + HI (*n* = 8)	–	6 h and 1 wk	Yes
*Innate defence regulator proteins*										
[60]	Mouse pups, P9	LPS, 0.3 mg/kg + HI CAL, 10% O_2_, 20 min	Innate defence regulator protein (IDR)-1018, 8 µg/g, i.p	3 h after insult	–	M + F, Ns not reported	↓ % White and grey matter tissue loss ↓ Injury scores in hippocampus, striatum, and thalamus LPS + HI + IDR1018 (*n* = 11) vs. LPS + HI + veh (*n* = 9)	–	1 wk	Yes
*Lipoxin A4*										
[72]	Rat pups, P7	HI, CAL, 8% O_2_, 2.5 h	LXA4 10 mg/kg, ICV	1 h after insult	Ambient T: 37.5 °C during HI	M + F, Ns not reported	↓% brain infarct area ↓Brain TNF and IL-6 protein HI + LXA4, (*n* = 3) vs. HI + veh (*n* = 3)	↑Motor function and cognition HI + LXA4 (*n* = 8) vs. HI + veh (*n* = 8)	Pathology: 1 day Function: 3 wks	Yes
*Cytokine IL-35 targeted therapies*										
[73]	Rat pups, P7	HI, CAO, 8% O_2_, 2 h	IL-35, 20 μg/g, i.v	Immediately and 1 d after insult	Ambient T: 37 ℃ during HI	–	↓Infarct volume ↓IL1β and TNFA mRNA in the cortex ↑Arg1, CD206, and YM-1 gene expression in the cortex of HI + IL-35 (*n* = 6) vs. HI + veh (*n* = 6)	↓Neurological deficit score HI + IL-35 (*n* = 8) vs. HI + veh (*n* = 8)	2 d	Yes
*Melanocortin receptor 1 agonists*										
[74]	Rat pups, P10	HI, CAL, 8% O_2_, 2.5 h	BMS-470539, 50 μg/kg OR 160 μg/kg, OR 500 μg/kg, i.n.,	1 h after insult	Ambient T: 37 ℃ during HI	–	↓% Infarct area HI + BMS470539 (160 and 500 μg/kg groups, *n* = 6/group) vs. HI + veh (*n* = 6)	↑Sensory motor function HI + BMS470539 (160 and 500 μg/kg groups, *n* = 6/group) vs. HI + veh (*n* = 6)	2 d	Yes
[74]	Rat pups, P10	HI, CAL, 8% O_2_, 2.5 h	BMS-470539, 160 μg/kg, i.n.,	1 h after insult	Ambient T: 37 ℃ during HI	–	↓ % tissue loss HI + BMS470539 (*n* = 8) vs. HI + veh (*n* = 8)	↑Sensory motor function HI + BMS470539 (*n* = 8) vs. HI + veh (*n* = 8)	4 wks	Yes
*COX2 inhibitors*										
[45]	Piglets, P1	Spontaneous IUGR	Ibuprofen, 20 mg/kg 24 h and 10 mg/kg on days 2 and 3, oral	1, 2 and 3 d after insult	–	M + F	↓IBA1 and GFAP + cells in IGWM and SCWM ↑Myelination ↑Neuron survival in cortex IUGR + ibu (*n* = 6) vs. IUGR + veh (*n* = 6)	–	3 d	Yes
[75]	Rat pups, P3	HI, CAO, 6% O_2_, 30 min	Ibuprofen, 100 mg/kg, s.c., and 50 mg/kg every 24 h	2 h after insult	Ambient T: 37 ℃ during HI	–	↓IBA + activation and IL-1β in frontal cortex and thalamus in HI + ibu (*n* = 8) vs HI + veh (*n* = 7)	–	1 wk	Yes
[53]	Rat pups, P5	LPS, 2 mg/kg, i.p	Celecoxib, 20 mg/kg, i.p	5 min after insult	Core T: 29.4–33.1 ℃	M + F, Ns not reported	↑Survival of progenitor OLs in the cingulum ↓Apoptosis, ↓IBA1 and GFAP + cells in cingulum and cortex LPS + Cel (*n* = 8) vs. LPS + veh (*n* = 8)	↑ Motor function LPS + Cel (*n* = 8) vs. LPS + veh (*n* = 8)	1 d	Yes
*Colony stimulating factors*										
[76]	Rat pups, P10	HI, Right CAL, 8% O_2_, 2.5 h	Granulocyte stimulating factor (G-CSF), 50 µg/kg, i.p	1 h after insult	Ambient T: 37 °C during HI	M + F, Ns not reported	↑Blood brain barrier integrity ↓NF- κB in whole brain HI + GCSF (*n* = 6) vs. vs. HI + veh (*n* = 6)	–	2 d	Yes
[77]	Rat pups, P10	HI, CAL, 8% O_2_, 2.5 h	Colony stimulating factor-1 (CSF-1), 80 μg/kg, i.n.,	1 and 24 h after insult	Core T: 37 °C during HI	–	↓%Infarct area ↓Brain oedema HI + CSF-1 (*n* = 4) vs. HI + veh (*n* = 4)	–	2 d	Yes
[77]	Rat pups, P10	HI, Right CAL, 8% O_2_, 2.5 h	CSF-1, 80 μg/kg, i.n	1 and 24 h after insult	Core T: 37 °C during HI	–	↓Tissue loss HI + CSF-1 (*n* = 8) vs. HI + veh (*n* = 8)	↑Sensorimotor function and ↑cognition HI + CSF-1(*n* = 6) vs. HI + veh (*n* = 6)	4 wks	Yes
*Cytokine TNF-targeted therapies*										
[52]	Rat pups, P3	LPS, 2 mg/kg, i.p	Etanercept, 5 mg/kg, i.p	Immediately after insult	Ambient T: 22 °C	M + F, Ns not reported	↑NG2 and O4 + cells and MBP ↓TUNEL and IBA1 + cells in cingulum LPS + etan (*n* = 6) vs. LPS + veh (*n* = 6)	–	1 d	Yes
[56]	Fetal sheep, 0.7 GA	i.v. LPS infusion + i.v. LPS boluses at 2, 3, and 4 d	Etanercept, 2 doses, 5 mg/kg infused over 30 min, 48 h apart, i.v	Immediately after insult	–	M + F	↓Iba1, GFAP, and TNF + cells and ↑ % mature oligodendrocytes in white matter LPS + etan (*n* = 8) vs. LPS + veh (*n* = 8)	↑EEG power in LPS + etan (*n* = 8) vs. LPS + veh (*n* = 8)	10 d	Yes
[40]	Fetal sheep, 0.7 GA	Umbilical cord occlusion, 25 min,	Etanercept, 1 mg, ICV	3 d after insult	–	M + F	↑ Olig2 and CC1 + cells ↑ MBP density in white matter HI + etan (*n* = 9) vs. HI + veh (*n* = 9)	–	3 wks	Yes
*Cytokine IL-1-targeted therapies*										
[49]	Fetal mice, E15	Maternal LPS, 200 μg, i.u	Anakinra, 10 mg/kg, maternal i.p	30 min before insult	–	–	↑Phos-nNOS ↑NMDA R1mRNA in the cortex LPS + IL-1Ra v LPS + veh (ns not reported)	–	4–6 h	Yes
[46]	Fetal mice, E16	Maternal IL-1β, 1 μg, i.u	Anakinra, 4 mg/kg, maternal s.c	30 min before insult	–	M + F	↓Microvascular degeneration vs. control in cortex, cingulum and hippocampus IL-1Ra + IL-1β (*n* = 8) vs veh + IL-1β (*n* = 8)	↑Visual evoked potentials IL-1Ra + IL-1β (*n* = 6) vs veh + IL-1β (*n* = 6)	Pathology: 15 d Function: 30 d	Yes
[46]	Fetal mice, E16	Maternal IL-1β, 1 μg, i.u.,	101.10, 1 mg/kg, maternal s.c	30 min before insult	–	M + F	↓ Cortical microvascular degeneration vs. control in cortex, cingulum and hippocampus IL-1Ra + IL-1β (*n* = 8) vs veh + IL-1β (*n* = 8)	Visual evoked potentials 101.10 + IL-1β (*n* = 6) vs veh + IL-1β (*n* = 6)	Pathology: 15 d Function: 30 d	Yes
[50]	Fetal rats, E20	Maternal LPS, 200 μg, i.p	Anakinra, 10 mg/kg, every 12 h from P1-9	Immediately after insult	–	M + F	↑Myelin density in the internal capsule ↑DCX + cells in hippocampal dentate gyrus ↓IBA1 + cells in CC and cingulum LPS + IL-1Ra (*n* = 10–13) vs LPS + veh (*n* = 10–13)	↑Motor function ↓Anxiety-like behaviour in LPS + IL-1Ra (*n* = 10–13) vs LPS + veh (*n* = 10–13)	40 d	Yes
[50]	Rat pups, P1	HI, CAL, 8% O_2_, 3.5 h	Anakinra, 10 mg/kg, every 12 h from P1-9	Immediately after insult	Ambient T: 37 °C during HI	M + F	↑Myelin density in the internal capsule ↑DCX + cells in hippocampal dentate gyrus ↓IBA1 + cells in CC and cingulum HI + IL-1Ra (*n* = 12–16) vs HI + veh (*n* = 12–16)	↑Motor function and ↓Anxiety-like behaviour in HI + IL-1Ra (*n* = 12–16) vs HI + veh (*n* = 12–16)	40 d	Yes
[50]	Fetal rats, E20,	Maternal LPS, 200 μg, i.p., + HI, CAL, 8% O_2_, 3.5 h	Anakinra, 10 mg/kg, every 12 h from P1-9	Immediately after insult	Ambient T: 37 °C during HI	M + F	No improvement in myelin density in the internal capsule ↑DCX + cells in hippocampal dentate gyrus in LPS + HI + IL-1Ra (*n* = 10–13) vs LPS + HI + veh (*n* = 10–13)	↑Motor function and ↓Anxiety-like behaviour in LPS + HI + IL-1Ra (*n* = 10–13) vs LPS + HI + veh (*n* = 10–13)	40 d	Yes
[51]	Rat pups, P3	LPS, 16 µg/µL, i.c	Anakinra, 2 mg/kg, i.p	5 min, then 6 and 22 h after insult	–	M	↓Hippocampal markers of neuronal integrity, excitatory amino acids, and membrane integrity LPS + veh (*n* = 16) vs. sham (*n* = 17) ↓ AD in corpus callosum and cingulum LPS vs. sham. ↓Fractin in corpus callosum and cingulum LPS + veh (*n* = 16) vs. sham (*n* = 17). No differences between LPS + IL-1Ra (*n* = 15) and sham	–	1 d	Yes
[39]	Fetal sheep, 0.85 GA	HI, bilateral CAO, 30 min	Anti- IL-1β monoclonal antibody, 7.7 mg/kg, i.v	15 min and 4 h after insult	–	–	↑Anti IL-1β mAb in brain parenchyma HI + IL-1β mAb (*n* = 5) vs. HI + veh (*n* = 5)	–	1 d	Yes
[38]	Fetal sheep, 0.85 GA	HI, bilateral CAO, 30 min	Anti- IL-1β monoclonal antibody, 5.1 mg/kg, i.v	15 min after insult	–	–	↓ ApopTag in non-neuronal cells ↓Caspase-3 activity ↓Global pathology score in the cortex of HI + IL-1β mAb (*n* = 10–12) vs. HI + veh (*n* = 10–14)	–	1 d	Yes
[55]	Fetal sheep, 0.85 GA	LPS, 400 µg, 800 µg, and 1200 µg, i.v	Anakinra, 13 mg/kg, i.v,	1 h after insult	–	M + F	↑Olig2 + cells ↓Caspase + cells ↓IBA1 + cells in white matter of LPS + IL-1Ra (*n* = 9) vs. LPS + veh (*n* = 8)	↑EEG power LPS + IL-1Ra (*n* = 9) vs. LPS + veh (*n* = 8)	4 d	Yes

### Preclinical models of neuroinflammation

Fetal or neonatal rodents (rats or mice) were the predominant species used (*n* = 47 studies). Eight studies used fetal rodents from embryonic days 15–20, broadly corresponding to the neural development of human infants at < 22 weeks of gestation [33, 34]. The 35 postnatal rodent studies ranged from postnatal days (P) 0–11. Eight studies used rodents between P 0–6, which is broadly comparable to human brain development at 22–32 weeks of gestation. Fifteen studies used rodents at P7, which is comparable to the preterm human brain at approximately 30–34 weeks. Sixteen studies used rodents at P 9–11, which is broadly comparable to human brain development at term [33, 34]. There were 12 large animal studies: six studies used fetal sheep at 0.7 of gestation, which is comparable to the preterm human brain at approximately 30 weeks of gestation [35, 36]. One used term neonatal piglets (postnatal day 1) and 5 used fetal sheep at 0.8–0.9 of term gestation; these ages are comparable to neural maturation in the term human brain [35, 36].

**Fig. 3:**
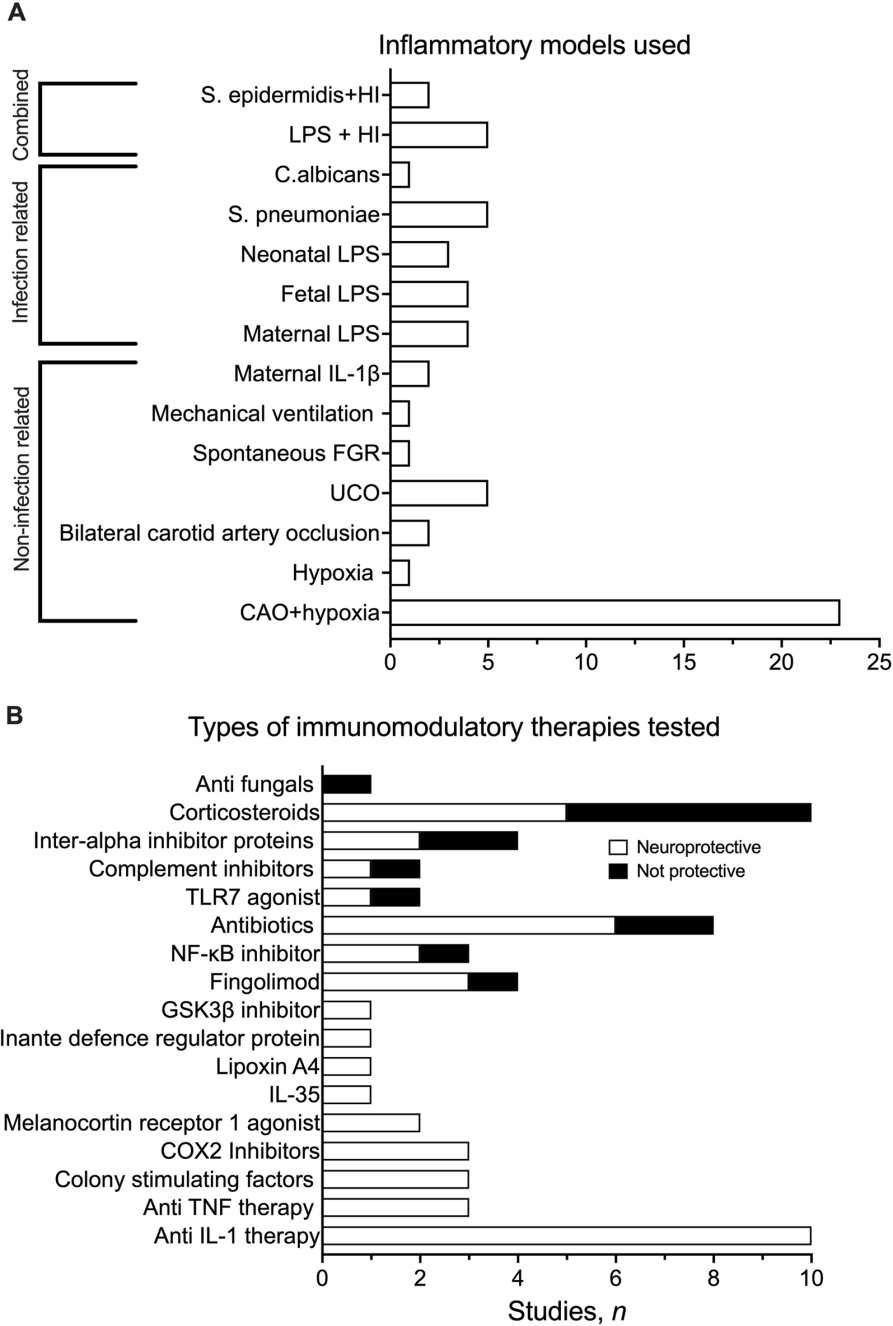
**A** Number of studies (*n*) which promoted inflammation using infection related, non-infection related and combined infection and non-infection related techniques and whether they showed the intervention to be neuroprotective (white) or not neuroprotective (black). **B** The number of studies (*n*) that showed neuroprotective outcomes (white) versus the number of studies that were not protective (black) for each therapy

Fourteen individual methods of causing inflammatory injury were identified (Fig. 3A). Studies were divided into three categories: inflammation initiated by pathogen-associated molecular patterns (infection-related inflammation, *n* = 17), inflammation initiated without pathogen-associated molecular patterns (non-infection related inflammation, *n* = 35), and combined infection- and not infection-related inflammation (*n* = 7). Twenty-three studies provoked neuroinflammation using the Rice–Vannucci model of carotid artery ligation followed by a period of moderate hypoxia. One study used neonatal hypoxia [37], and two studies used bilateral carotid artery occlusion [38, 39]. Five studies used umbilical cord occlusion in fetal sheep [40–44], one study used spontaneous fetal growth restriction in neonatal piglets [45] and 2 studies induced fetal inflammation by injecting IL-1β between the fetal membranes [46]. Eleven studies induced inflammation using the Gram-negative bacterial cell wall component lipopolysaccharide (LPS); four administered LPS maternally (using either intrauterine or intraperitoneal injection) [46–50] and seven infused LPS directly to the fetus or newborn using either single intracerebral, intracisternal or intraperitoneal injection to the neonate [51–54] or repeated fetal i.v. LPS infusions [55, 56]. Five studies used intracisternal injection of live *S. pneumoniae* to the newborn [57]. Seven studies combined either intraperitoneal injection of live *S. epidermidis* (*n* = 2) [58] or LPS (*n* = 5) with neonatal hypoxia–ischaemia [50, 59–61].

### Therapeutic doses, regimens, outcomes, and survival times

Twenty anti-inflammatory/immunomodulatory therapies in 17 categories were investigated. A description of each therapy, a summary of the number of studies that reported neuroprotection vs. no protection for each therapy are outlined in Table 2 and Fig. 3B, respectively.

**Table 2:** List of immunomodulatory therapies analysed in this review, their Therapeutic Goods Administration (TGA)/Food and Drug Administration (FDA) approval status and their mechanism of action

Reference(s)	Therapeutic	TGA/FDA approval	Mechanism
*Antifungals*			
[61]	Fluconazole	Yes/yes	Selective inhibitor of fungal cell wall synthesis
*Corticosteroids*			
[43, 44, 54, 62, 63]	Dexamethasone	Yes/yes	A corticosteroid that acts on glucocorticoid receptors which suppresses neutrophil migration, macrophage activation and lymphocyte proliferation and decreases permeability of capillaries. More rapid onset and shorter duration of action than betamethasone
[62]	Hydrocortisone	Yes/yes	A corticosteroid that acts on glucocorticoid receptors which suppresses neutrophil migration, macrophage activation and lymphocyte proliferation and decreases permeability of capillaries. Less potent and shorter acting than dexamethasone
[54, 64]	Betamethasone	Yes/yes	A corticosteroid that acts on glucocorticoid receptors which suppresses neutrophil migration, macrophage activation and lymphocyte proliferation and decreases permeability of capillaries. More potent and longer lasting than dexamethasone and hydrocortisone
*Inter-alpha inhibitor proteins*			
[65, 66]	Human plasma derived inter-alpha inhibitor proteins	No/no	Endogenous human plasma proteins that block the release of serine proteases protecting cells from cytotoxicity
*Complement inhibitors*			
[67]	RLS-0071	No/no	An amino acid peptide that binds to the C1q compliment protein preventing downstream signalling of the compliment pathway
*TLR7 agonist*			
[41, 42]	(Gardiquimod) GDQ	No/no	An imidazoquinoline analogue that induces the activation of NF-κB in cells expressing human or mouse TLR7
*Antibiotics*			
[57]	Ceftriaxone	Yes/yes	A broad-spectrum cephalosporin antibiotic that inhibits the mucopeptide synthesis in the bacterial cell wall
[57]	Daptomycin	Yes/yes	A broad-spectrum cyclic lipopeptide antibiotic against Gram-positive bacteria. Disrupts bacterial cell membrane function
[58]	Vancomycin	Yes/yes	A glycopeptide antibiotic against Gram-positive bacteria. Inhibits cell wall biosynthesis
[68]	Doxycycline	Yes/yes	A tetracycline antibiotic that inhibits bacterial protein synthesis
*Methylxanthines*			
[58]	Pentoxifylline	Yes/yes	A methylxanthine derivative that lowers blood viscosity by increasing erythrocyte flexibility, reducing plasma fibrinogen, inhibiting neutrophil activation, and suppressing erythrocyte/platelet aggregation
*NF-kB inhibitors*			
[59]	Tat-NBD peptide	No/no	A 22 amino acid peptide that inhibits NF-κB signalling by penetrating the cell and blocking the NF-κB essential modifier (NEMO)
*Fingolimod*			
[37, 47, 48, 70]	Fingolimod (FTY720)	Yes/yes	A sphingosine 1-phosphate (S1P) receptor agonist that causes lymphocytes to be sequestered to the lymph nodes
*GSK3β inhibitor*			
[71]	SB216763	No/no	Selectively inhibits the activity of GSK-3α and GSK-3β, preventing PI3-kinase induced cell death
*Innate defence regulator proteins*			
[60]	IDR-1018	No/no	A synthetic 12 amino acid antibiofilm peptide that selectively binds to the nucleotide (p)ppGpp inhibiting bacterial function
*Lipoxin A4*			
[72]	LXA4	No/no	A metabolite of arachidonic acid that stimulates the bacteria-killing capacity of leukocytes, inhibit neutrophil infiltration and pro-inflammatory cytokine and chemokine production via inhibition of NF-κB and activator protein 1
*Cytokine IL-35 targeted therapies*			
[73]	Recombinant human IL-35	No/no	An anti-inflammatory cytokine that induces regulatory T and B lymphocytes
*Melanocortin 1 receptor agonists*			
[74]	BMS-470539	No/no	A small molecule that acts as a selective agonist of the melanocortin 1 receptor promoting downstream signalling
*COX2 inhibitors*			
[45, 75]	Ibuprofen	Yes/yes	A non-steroidal anti-inflammatory that non selectively inhibits COX1 and COX2 to reduce prostaglandin synthesis
[53]	Celecoxib	Yes/yes	A non-steroidal anti-inflammatory drug (NSAID) that selectively inhibits COX2 and decreases prostaglandin synthesis
*Granulocyte colony-stimulating factor*			
[76]	Human G-CSF produced by recombinant DNA technology	No/no	An endogenous lipoxygenase-derived eicosanoid mediator that suppresses leukocytes and inhibits production of pro-inflammatory cytokines
*Colony stimulating factor 1*			
[77]	Rh-CSF1	No/no	Recombinant human growth factor of CSF1 that leads to the recruitment of CSF1R expressing cells including macrophages, monocytes and dendritic cells
*Cytokine TNF targeted therapies*			
[40, 52, 56]	Etanercept	Yes/yes	A soluble TNF receptor that sequesters TNF to prevent it from interacting with endogenous TNF receptors
*Cytokine IL-1 targeted therapies*			
[38, 39]	Mouse anti- ovine-IL-1β, monoclonal antibodies	No/no	A mouse-anti-ovine IL-1β monoclonal antibody that binds to ovine IL-1β and neutralises inflammation by blocking IL-1β from interacting with the IL-1β receptors
[46, 49–51, 55]	Anakinra	Yes/yes	A recombinant human IL-1 receptor antagonist that competitively binds to the IL-1 receptor inhibiting the activity of IL-1α and IL-1β
[46]	101.10 (Rytvela)	No/no	An allosteric IL-1 receptor peptide antagonist that selectively binds to the IL-1 receptor inhibiting the activity of IL-1α and IL-1β

**Fig. 4:**
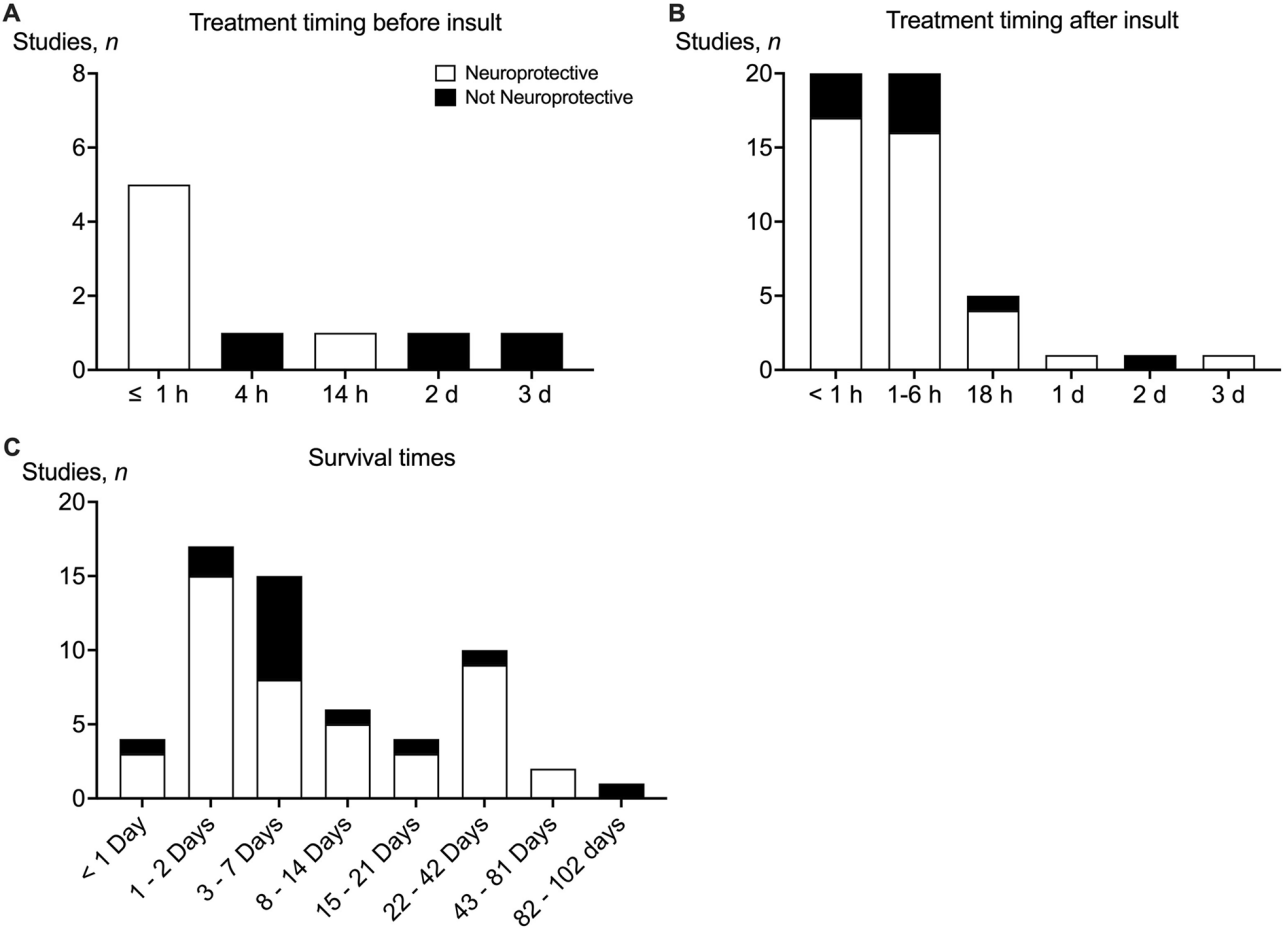
**A** The number of studies (*n*) that showed neuroprotection (white) or no protection (black) after administering the treatment at ≤ 1 h, 4 h, 14 h, 2 d or 3 d before the insult. **B** The number of studies (*n*) that showed neuroprotection (white) or no protection (black) after administering the treatment at < 1 h, 1–6 h, 18 h, 1 d, 2 d, or 3 d after the insult. **C** The number of studies (*n*) that showed neuroprotection (white) or no protection (black) stratified by survival time after the insult

Nine studies started the intervention before the insult (Fig. 4A), and 50 studies administered the intervention after the insult. For the latter approach, most studies started treatment either within the first hour (*n* = 20/50, 40%) or between 1 and 6 h after the insult (*n* = 21/50, 42%) (Fig. 4B). Only 4 studies started treatment between 1 and 3 d after the insult, of which 3 reported neuroprotection and 1 reported increased injury after treatment (Fig. 4B). The treatment dose, regimen and survival times (Fig. 4C) varied markedly. The main outcomes for each therapy are described below and in Table 1, in order of least-to-most effective, according to the proportion of studies that reported no improvement or deleterious outcomes vs. those that showed improved outcomes, as indicated by brain histopathology or behavioural assessment.

One study administered the anti-fungal treatment fluconazole (Table 2) to the fetus 2 days after exposure to intra-amniotic *Candida albicans* and showed increased neuroinflammation and oligodendrocyte loss (*P* < 0.05, Kruskal–Wallis with Dunnett’s post hoc test) [61] Table 1).

Ten studies tested corticosteroids (hydrocortisone, dexamethasone or betamethasone, Table 2). The less potent corticosteroid, hydrocortisone, in a dose of 10 µg given intracerebroventricularly 2 h after HI was associated with reduced infarct size at 2 days (*P* < 0.05, one-way ANOVA with Newman–Keuls post hoc) [62]. Similarly, reduced infarct size after 2 days was seen with 300 µg given intranasally 2 h after HI. However, protective or injurious effects were not seen with lower or higher intranasal doses (50–1000 µg) (*P* < 0.05, one-way ANOVA with Newman–Keuls post hoc) [62] (Table 1). Repeated i.p. doses of dexamethasone (range: 0.1–0.5 mg/kg) given 4 days before HI were associated with increased neuronal cell death after 1 day recovery (*P* < 0.05) [63]. A single intracerebral injection of dexamethasone or betamethasone given 1 h before LPS was associated with improved histological and behavioural outcomes (*P* < 0.05, one-way ANOVA with Tukey’s post hoc) [54]. However, a single intranasal dose of dexamethasone (0.1 µg) given 2 h after HI was not associated with improved outcomes after 2 days [62].

In preterm fetal sheep, a single 12 mg i.m. dose of maternal dexamethasone given either 4 h before or 15 min after global HI was associated with increased electrographic seizures (*P* < 0.05, repeated measures ANOVA with Fisher’s LSD post hoc) and increased white and grey matter injury after 7 days (*P* < 0.05, three-way ANOVA) [43, 44] (Table 1). Similarly, maternal betamethasone (11.2 mg at 48 and 24 h before preterm birth) was associated with increased inflammation, oxidative stress and vascular extravasation in neonatal lambs exposed to high tidal volume ventilation (*P* < 0.05, two-way ANOVA with Holm–Sidak post hoc test) [64] (Table 1).

Four studies investigated giving repeated doses of inter-alpha inhibitor proteins (a serine protease inhibitor; Table 2) (30 mg/kg i.p.) to the neonate. Two showed no improvement in histology (at 3 days) and behavioural outcomes (at ~ 16 weeks), respectively, with treatment started from one to six hours after HI (*P* < 0.05, one-way ANOVA) [65, 66]. In contrast, two studies showed reduced tissue loss (*P* < 0.05, one-way ANOVA with Fisher’s LSD post hoc) and improved memory at 3 days and ~ 13 weeks (*P* < 0.05, repeated measures ANOVA with Tukey’s post hoc), respectively, when treatment was started within the first hour after HI, although improved outcomes were only seen in male offspring [65, 66] (Table 1).

Two studies used the complement inhibitor RLS0071 (Table 2). Both studies gave single or repeated doses of 10 mg/kg i.p., starting 1 h after HI. One showed no improvement in histological outcomes after 2 days (*P* < 0.05, paired T-test and ANOVA) [67]. One showed reduced cortical infarct area 2 days after HI when complement inhibitor was combined with therapeutic hypothermia (compared to hypothermia alone) (*P* < 0.05, paired T test and ANOVA) [67] (Table 1).

Two studies used a toll-like receptor 7 (TLR7) agonist (gardiquimod (GDQ), Table 2) at a dose of 1.8 mg/kg via fetal intracerebroventricular infusion from 1 h after global HI. Improved neuronal and oligodendrocyte survival were seen 3 days after treatment (*P* < 0.05, two-way ANOVA with Fisher’s LSD post hoc) [41], whereas there was delayed onset of epileptiform discharges and no overall histological improvement after 7 days recovery (*P < 0.05, repeated measures ANOVA with Fisher’s LSD post hoc) [42] (Table 1).

Eight, studies tested antibiotics for induced bacterial infection. One study used a single i.p. dose of 15 mg/kg vancomycin (Table 2) given 2 min after neonatal *S. epidermidis* inoculation combined with HI. Treatment was associated with attenuated brain tissue loss 9 days later (*P* < 0.05, Kruskal–Wallis test with Dunn’s post hoc) [58]. In the same animal model, combining pentoxifylline (40 mg/kg i.p.) with vancomycin did not augment vancomycin-induced protection (*P* > 0.05, Kruskal–Wallis test with Dunn’s post hoc) [58] (Table 1). One study used 10 mg/kg doxycycline i.p. given 1 h after HI and showed reduced lesion size and neuronal loss after 42 days (*P* < 0.05, Mann–Whitney U test) [68]. One study administered ceftriaxone (Table 2) at a dose of 100 mg/kg i.p. 18 h after intracisternal *S. pneumonia* inoculation. Treatment was associated with increased neuronal loss after 42 h (*P* < 0.05, Mann–Whitney test), and reduced learning and memory after 3 weeks (*P* < 0.05, two-way ANOVA) [57] (Table 1). One study used 100 mg/kg ceftriaxone i.p. combined with the non-bacteriolytic antibiotic daptomycin 10 mg/kg s.c. given 18 h after intracisternal *S. pneumonia* inoculation and showed reduced cortical necrosis after 42 h (*P* < 0.05, Mann–Whitney test) [57] (Table 1). Three studies combined ceftriaxone i.p. with daptomycin 10 mg/kg s.c. and 2 doses of the matrix metalloprotease inhibitor trocade (75 mg/kg) given 24 h apart starting at 18 h after intracisternal *S. pneumonia* inoculation. This treatment regimen was associated with reduced hippocampal apoptosis and cortical necrosis after 42 h (*P* < 0.05, Mann–Whitney test), and improved hearing, learning and memory at 3 weeks (*P* < 0.05, two-way ANOVA) [57] (Table 1).

Three studies used a single dose of a nuclear factor kappa B (NF-*κ*B) inhibitor (Tat-NBD, Table 2) delivered intranasally to the neonate at a dose of 1.4 mg/kg 10 min after the insult. Two showed reduced tissue loss after 7 days in rat pups exposed to a combination of HI and LPS and one showed no improvement in histological outcomes in pups exposed to HI alone (*P* < 0.05, unpaired *t*-test or one-way ANOVA with Newman–Keuls post hoc) [59] (Table 1).

Four studies tested fingolimod (FTY720, Table 2), a sphingosine-1-phosphate receptor modulator [69]. Of these, two gave it antenatally to the mother, as a single dose of 1 mg/kg i.p. immediately or 30 min after maternal LPS-exposure and showed improved histological outcomes (reduced markers of inflammation in the white matter and cortex) after 6- and 4-h recovery, respectively (*P* < 0.05, Mann–Whitney test) [47, 48]. Two studies gave fingolimod to the neonate via single or repeated doses (0.3–1 mg/kg, i.p.). The 1 mg/kg dose was associated with worse histological outcomes (increased cortical tissue loss) compared to vehicle 7 days after HI (*P* < 0.05, unpaired *t*-test.), whereas 0.3 mg/kg was associated with reduced total seizure duration and improved behavioural outcomes at 7 weeks after HI (*P* < 0.05, two-way ANOVA with Tukey’s post hoc) [37, 70] (Table 1).

One study used repeated doses of a glycogen synthase kinase 3 β (GSK3β) inhibitor (SB216763, Table 2) at 10 mg/kg i.p. to the neonate from 14 h before the insult and showed reduced tissue loss at 7 days after HI (*P* < 0.05, one-way ANOVA with Holm–Sidak’s post hoc) [71] (Table 1).

One study gave an innate defence regulator protein 1018 (IDR-1018, Table 2) in a single dose (8 µg/g i.p.) to the neonate at 3 h after LPS + HI and showed reduced white and grey matter tissue loss 7 days after treatment (*P* < 0.05, *t* test) [60] (Table 1).

One study used single intracisternal infusion of lipoxin A4 (Table 2) at a dose of 10 mg/kg starting 1 h after HI and showed reduced infarct area and improved motor function and cognition at 24 h and 3 weeks, respectively (*P* < 0.05, one-way ANOVA with Tukey’s post hoc) [72] (Table 1).

Recombinant human IL-35 was administered i.v. to the neonate at the time of HI and 1 day later, reduced infarct volume was shown 2 days after treatment (*P* < 0.05, one-way ANOVA with Tukey’s post hoc) [73] (Table 1).

Two studies administered a single dose of a melanocortin receptor 1 agonist (BMS-470539, Table 2) intranasally at 1 h after HI [74]. The concentrations tested ranged from 50 µg/kg to 500 µg/kg, with survival times between 2 days and 4 weeks. Outcomes were dose dependent; 50 µg/kg did not improve outcomes, whereas 500 µg/kg and 160 µg/kg reduced infarct area and improved sensorimotor function at 2 days and 4 weeks, respectively (*P* < 0.05, one-way ANOVA or Student *t*-test with Tukey’s post hoc) (Table 1).

Three studies used cyclooxygenase 2 (COX2) inhibitors (ibuprofen and celecoxib; Table 2) administered to the neonate via single or repeated doses of 10–20 mg/kg from 5 min to 2 h (i.p.) after LPS exposure, or 1 day (oral) after delivery in a model of spontaneous growth restriction. One showed improved histological outcomes (reduced inflammation and improved white and grey matter integrity) and motor function after one day (*P* < 0.05 one-way ANOVA with Student–Newman–Keuls post hoc) [53], one showed reduced inflammation in the frontal cortex after 10 days (*P* < 0.05, *t*-test) [75], and one showed reduced white matter gliosis, improved myelination and neuronal survival after three days (*P* < 0.05, two-way ANOVA with Holm–Sidak post hoc) [45] (Table 1).

Three studies used either granulocyte (G-CSF, Table 2) or colony stimulating factor 1 (CSF-1/M-CSF, Table 2). When G-CSF was given as a single i.p. 50 µg/kg dose intraperitoneally at 1 h after HI, improved blood brain barrier integrity and reduced inflammation were reported after 2 days recovery (*P* < 0.05, one-way ANOVA with Tukey’s post hoc) [76]. CSF-1 was given via repeated doses of 80 µg/kg intranasally at 1 and 24 h after HI. Reduced and sensorimotor and cognitive function were shown after 2 days and 4 weeks recovery, respectively (*P* < 0.05, one-way ANOVA with Tukey’s post hoc) [77] (Table 1).

Etanercept, a soluble TNF receptor (Table 2) that inhibits TNF activity, was administered directly to the fetus or neonate in three studies. A single i.p. dose (5 mg/kg) was associated with improved white matter integrity 1 day after hypoxia–ischaemia (HI) (*P* < 0.05, ANOVA with Bonferroni post hoc) [52]. One study gave repeated doses of etanercept i.v. to fetal sheep (5 mg/kg) starting immediately after LPS exposure and one study administered it to the fetal sheep brain via repeated intracerebroventricular infusions (1 mg) starting from 3 days after HI. Both showed improved reduced neuroinflammation and reduced white matter injury (*P < 0.05, two-way ANOVA with Fisher’s LSD post hoc) and or reduced suppression of electroencephalogram power (repeated measures ANOVA with Fisher’s LSD post hoc) at 10 days and 3 weeks [40, 56] (Table 1).

For studies targeting IL-1, eight studies gave IL-1 receptor antagonists (anakinra or 101.10, Table 2) at doses of 1 to 13 mg/kg. Three treated prophylactically, i.e. starting before the insult, and used single dosing in fetal mice. All showed improved histological (reduced markers of neurotoxicity and improved microvascular integrity) (*P* < 0.05, Kruskal–Wallis one-way ANOVA) and functional outcomes (improved visual evoked potentials) (*P* < 0.05, Kruskal–Wallis one-way ANOVA with Dunn’s post-test) in the offspring when assessed at 4–6 h, and 15–30 days after the insult, respectively [46, 49] (Table 1). Three studies treated the neonate directly using repeated doses started immediately after the insult in postnatal mice exposed to maternal LPS and or neonatal hypoxia. All showed improved histological (*P* < 0.05, ANOVA with Newman–Keuls post hoc test) and functional outcomes (*P* < 0.05, unpaired *t*-test with Welch correction) 40 days later [50] (Table 1). One study gave three doses of anakinra between 5 min and 22 h after the insult to LPS-treated male rat pups and showed improved histological and MRI outcomes 1 day later (*P* < 0.05, Kruskal–Wallis tests with Dunn’s multiple comparisons) [51]. One study gave anakinra 1 h after progressive repeated LPS exposure in fetal sheep and showed both improved histological (*P* < 0.05, two-way ANOVA with Fisher’s LSD) and functional (improved electroencephalogram power, *P* < 0.05 two-way ANOVA with repeated measures) after 4 days [55]. Two studies gave one or two doses of 5.1 to 7.7 mg/kg to the fetus of a mouse anti-ovine IL-1β monoclonal antibody (Table 2) starting 15 min after the insult. Both showed improved histological outcomes (blood brain barrier penetration and reduced grey matter apoptosis) after 1-day recovery (*P* < 0.05, *P* < 0.05, one-way ANOVA with FSD post hoc) [38, 39] (Table 1).

### Temperature monitoring

**Fig. 5:**
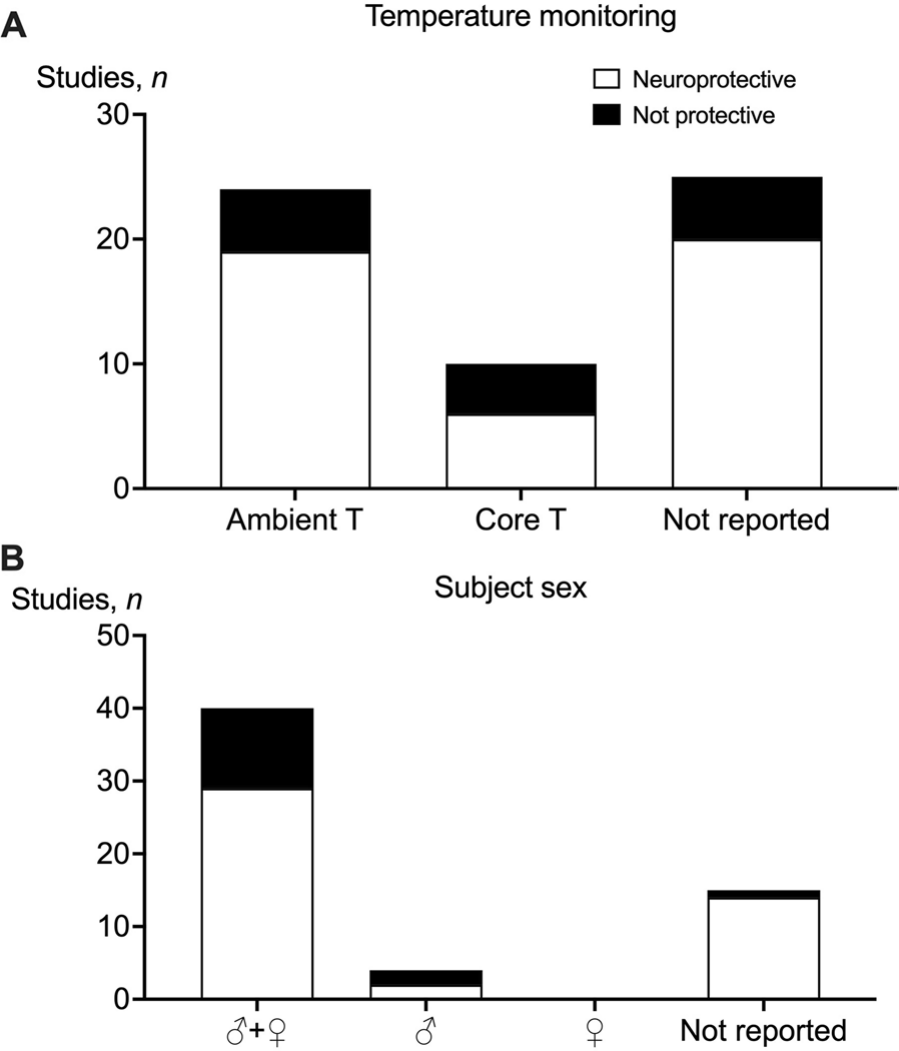
**A** The number of studies (*n*) that showed neuroprotection (white) or no protection (black) and monitored ambient temperature, core temperature, or did not report temperature monitoring. **B** The number of studies (*n*) that showed neuroprotection (white) or no protection (black) which reported outcomes in both males and females (♂ + ♀), males only (♂), females only (♀), or did not report the sex of the subjects

Ten out of 59 studies (16%) reported monitoring core temperature during the study (Table 1, Fig. 5A). Of these, 5 reported maintaining core temperature during the insult (HI) but not during recovery. One study reported temporal core temperature data throughout the experimental period [58]. Twenty-three studies reported maintaining ambient air temperature (range: 28–38 ℃) during the study period, 18/23 (78%) reported neuroprotection. Twenty five studies did not report temperature monitoring as part of their study protocol, however 11/25 studies (44%) were conducted in fetal sheep, where fetal core temperature is maintained in utero between 39.0 and 39.5 ℃ by the intrauterine environment [78, 79]. An overview of type of temperature control for the studies included can be seen in Fig. 5A.

### Subject sex

Forty-one out of 59 studies (69%) reported outcomes in both sexes, but 11 of these studies did not report numbers or ratios of males and females (Table 1, Fig. 5B). Four out of 59 studies reported outcomes in males only [51, 63, 66]. Of these, 2 reported improved outcomes, one reported no improvement and one reported worse outcomes with treatment. Fifteen out of 59 studies (25%) did not report the sex of the subjects, of these, 14/15 studies showed improved outcomes (Fig. 5B).

### Study bias

The SYRCLE RoB tool [32] was used to measure risk of study bias (Table 3). Thirty out of 40 papers stated that allocation to groups was random, although only two papers gave specific details relating to how the randomisation was performed [47, 74]. Nine out of 40 papers reported the baseline characteristics of the groups analysed. No studies explicitly reported randomly housing animals during the experiment or noted whether the caregivers and examiners were blinded to treatment groups. Seventeen out of the 40 papers (42%) did not report blinding of the assessor/s during the analysis, while one paper reported conducting a random outcome assessment [65]. Seventeen out of 40 papers (42%) did not address incomplete outcome data and were therefore at risk of attrition bias. All papers appeared to be free from selective outcome reporting (Table 3).

**Table 3:** SYRCLE risk of bias assessment for included studies

References	Selection bias			Performance bias		Detection bias		Attrition bias	Reporting bias	Free from other bias?
	Random sequence generation	Groups similar at baseline	Allocation concealment	Animals random housing	Blinding of caregivers and/or examiners	Random outcome assessment	Blinding of outcome assessor	Incomplete outcome data addressed	Free from selective outcome reporting	
[61]	Unclear	Unclear	Yes	Unclear	Unclear	Unclear	Yes	Unclear	Yes	No
[54]	Unclear	Unclear	Unclear	Unclear	Unclear	Unclear	Yes	Unclear	Yes	No
[63]	Unclear	Unclear	Unclear	Unclear	Unclear	Unclear	Unclear	Unclear	Yes	Yes
[62]	Unclear	Unclear	Yes	Unclear	Unclear	Unclear	Unclear	Unclear	Yes	Yes
[43]	Unclear	Yes	Yes	Unclear	Unclear	Unclear	Yes	Unclear	Yes	Yes
[44]	Unclear	Yes	Yes	Unclear	Unclear	Unclear	Yes	Yes	Yes	Yes
[64]	Unclear	Unclear	Yes	Unclear	Unclear	Unclear	Yes	Yes	Yes	Yes
[65]	Unclear	Unclear	Yes	Unclear	Unclear	Yes	Unclear	Yes	Yes	Yes
[66]	Unclear	Unclear	Yes	Unclear	Unclear	Unclear	Unclear	Yes	Yes	Yes
[67]	Unclear	Unclear	Yes	Unclear	Unclear	Unclear	Yes	Unclear	Yes	Yes
[41]	Unclear	Yes	Yes	Unclear	Unclear	Unclear	Yes	Yes	Yes	Yes
[42]	Unclear	Yes	Yes	Unclear	Unclear	Unclear	Yes	Yes	Yes	Yes
[58]	Unclear	Unclear	Yes	Unclear	Unclear	Unclear	Yes	Unclear	Yes	Yes
[68]	Unclear	Unclear	Yes	Unclear	Unclear	Unclear	Unclear	Yes	Yes	Yes
[57]	Unclear	Unclear	Yes	Unclear	Unclear	Unclear	Yes	Unclear	Yes	Yes
[59]	Unclear	Unclear	Yes	Unclear	Unclear	Unclear	Unclear	Unclear	Yes	Yes
[47]	Yes	Unclear	Yes	Unclear	Unclear	Unclear	Unclear	Unclear	Yes	Yes
[48]	Unclear	Unclear	Unclear	Unclear	Unclear	Unclear	Yes	Unclear	Yes	No
[70]	Unclear	Unclear	Yes	Unclear	Unclear	Unclear	Unclear	Yes	Yes	Yes
[37]	Unclear	Unclear	Yes	Unclear	Unclear	Unclear	Unclear	Yes	Yes	Yes
[71]	Unclear	Unclear	Unclear	Unclear	Unclear	Unclear	Yes	Unclear	Yes	Yes
[60]	Unclear	Unclear	Unclear	Unclear	Unclear	Unclear	Yes	Unclear	Yes	No
[72]	Unclear	Unclear	Yes	Unclear	Unclear	Unclear	Unclear	Yes	Yes	Yes
[73]	Unclear	Unclear	Yes	Unclear	Unclear	Unclear	Yes	Yes	Yes	Yes
[74]	Yes	Unclear	Yes	Unclear	Unclear	Unclear	Yes	Yes	Yes	Yes
[45]	Unclear	Unclear	Unclear	Unclear	Unclear	Unclear	Yes	Yes	Yes	Yes
[75]	Unclear	Unclear	Yes	Unclear	Unclear	Unclear	Unclear	Yes	Yes	Yes
[53]	Unclear	Unclear	Unclear	Unclear	Unclear	Unclear	Unclear	Unclear	Yes	Yes
[76]	Unclear	Unclear	Unclear	Unclear	Unclear	Unclear	Unclear	Yes	Yes	Yes
[77]	Unclear	Unclear	Yes	Unclear	Unclear	Unclear	Yes	Yes	Yes	Yes
[52]	Unclear	Unclear	Unclear	Unclear	Unclear	Unclear	Unclear	Unclear	Yes	Yes
[56]	Unclear	Yes	Yes	Unclear	Unclear	Unclear	Yes	Yes	Yes	Yes
[40]	Unclear	Yes	Yes	Unclear	Unclear	Unclear	Yes	Yes	Yes	Yes
[49]	Unclear	Unclear	Yes	Unclear	Unclear	Unclear	Unclear	Unclear	Yes	No
[50]	Unclear	Yes	Yes	Unclear	Unclear	Unclear	Yes	Yes	Yes	Yes
[46]	Unclear	Unclear	Unclear	Unclear	Unclear	Unclear	Yes	Yes	Yes	Yes
[51]	Unclear	Unclear	Yes	Unclear	Unclear	Unclear	Unclear	Yes	Yes	Yes
[39]	Unclear	Yes	Yes	Unclear	Unclear	Unclear	Unclear	Yes	Yes	Yes
[38]	Unclear	Yes	Yes	Unclear	Unclear	Unclear	Yes	Unclear	Yes	Yes
[55]	Unclear	Yes	Yes	Unclear	Unclear	Unclear	Yes	Yes	Yes	Yes

## Discussion

Perinatal inflammation is a major cause of neurodevelopmental impairments in preterm and term infants [25, 26]. Developing effective therapeutic interventions for the ‘at risk’ fetus or neonate requires that we improve our understanding of the pathophysiological mechanisms that lead to neurodevelopmental impairments, identify therapeutic targets, and test pharmacological interventions in a translational research pipeline that incorporates high quality small and large animal trials. In this systematic review, we set out to identify which immunomodulatory interventions have been trialled between 2012 and 2023 for inflammation-induced brain injury and determine key knowledge gaps in the literature that need to be addressed in animal studies before progressing potential therapies into human trials for perinatal neuroprotection.

### Modelling perinatal infection/inflammation

There is compelling evidence that both mild and moderate-to-severe HIE and infection/inflammation are highly associated with microgliosis and activation of distinct inflammatory pathways in the peripheral and central nervous system, as previously reviewed [19, 26, 80]. Most of the studies surveyed here (59%) used models of ‘non-infection’ related inflammation (hypoxia with or without ischaemia). A few studies modelled ‘infection’ related inflammation (28%) or combined ‘infection and non-infection’ related insults (12%). None tested interventions in the setting of Gram-positive infection, such as mycoplasmas (e.g. Ureaplasma spp.), which are among the most common bacterial isolates in pregnancies complicated by chorioamnionitis (fetal infection/inflammation), preterm birth [81], and neurodevelopmental impairment. For example, amniotic fluid cultures that are positive for Ureaplasma urealyticum are associated with a higher risk of adverse psychomotor development, abnormal neurological outcome and a higher risk of cerebral palsy at 2 years of age compared to patients with negative amniotic fluid cultures [82].

None of the studies surveyed used polymicrobial models of inflammation. There is emerging evidence that multiple bacteria and viruses reside in the placenta and amniotic fluid, raising the possibility that, at least in some cases, there may be a polymicrobial aetiology to perinatal infection/inflammation-induced impairments in brain development [83–87]. This concept is supported by studies in animal models that show combining viral and bacterial inflammation in pregnant mice is associated with increased rates of preterm birth, tissue inflammation and necrosis relative to either inflammatory stimulus alone [88, 89]. Furthermore, few studies modelled repeated fetal or neonatal infection/inflammation. Repeated infections occur in approximately two thirds of preterm infants ≤ 30 weeks of gestation and are associated with an increased risk of white matter abnormalities and mortality [90, 91]. Another consideration is that none of the studies surveyed tested immunomodulators in models of viral infection. This highlights another important knowledge gap given the strong association between congenital infections with viruses, such as cytomegalovirus herpes simplex virus type 1 and severe acute respiratory syndrome coronavirus 2, and long-term neurological sequelae [92–95].

### Controlling for iatrogenic hypo/hyperthermia

Most publications (*n* = 47/59, 79%) used neonatal rodents. Rigorous studies in neonatal rodents offer many advantages for neuroprotection research, as previously highlighted [96]. However, due to their small body mass relative to surface area, lack of subcutaneous fat, naked skin and limited shivering response, neonatal rats produce less heat and lose more body heat than adults [96, 97]. These factors make them functionally poikilothermic and susceptible to rapid changes in body and brain temperature during changes in environmental temperature [98]. Small changes in body temperature are known to affect neurological outcomes in animal and human studies [96, 99, 100]. Furthermore, as previously reviewed, neuroprotective effects of various pharmacological interventions, including anaesthetics, can be confounded by drug-induced hypothermia mediated by increased heat loss [100]. Conversely, neuroprotection can be masked by delayed hyperthermia [101, 102]. Thus, care is required to ensure that iatrogenic changes in body temperature do not occur to ensure that outcomes are not confounded by unappreciated changes in body temperature or environmental conditions.

Of concern, only 16% of studies published since 2012 measured core temperature; half of these studies measured core temperature during the insult, and one explicitly reported temperature data after treatment [58]. Most of the studies measured environmental temperature which ranged from 28–38 ℃ (− 4 to 0 ℃ below core temperature). We identified 4 studies that used maternal LPS exposure to model antenatal infection/inflammation, all reported modest improvements in neurological outcomes, but none monitored maternal body temperature. Two of these studies administered fingolimod, a peripheral vasodilator [103], to the mother. One study did not state whether temperature was maintained, the other reported maintaining ambient temperature between 21 and 22 ℃. This is an important consideration since maternal LPS exposure is commonly associated with pyrexia. Intrapartum fever is associated with adverse neonatal outcomes and increased risk of cerebral palsy and neonatal encephalopathy [104], likely mediated by a combination of increased release of oxygen free radicals and excitatory neurotransmitters, enhanced glutamate toxicity on neurons and glia, blood brain barrier dysfunction and proteolysis [105]. Thus, it is not possible to know whether neuroprotective effects of fingolimod were mediated by iatrogenic hypothermia in the pregnant dams or direct anti-inflammatory effects of fingolimod.

Of the 25 studies that did not report controlling body temperature, 10 were conducted in fetal sheep. A major advantage of testing potential neuroprotectants in fetal sheep is that their body temperature is regulated by the pregnant ewe and therefore unless the ewe is febrile, fetal core temperature is highly stable [78, 79]. Collectively these observations highlight the need for animal studies to improve core temperature monitoring throughout the experimental period to ensure that outcomes of preclinical drug trials are not confounded by fluctuations in maternal, fetal or neonatal body temperature.

### Limitations of current immunomodulatory therapies: corticosteroids and antibiotics

Currently there are no clinically proven treatments to prevent infection/inflammation related brain injury. Of the immunomodulatory interventions identified in this systematic review corticosteroids and antibiotics are among the most routinely used interventions in perinatal medicine. In our analysis, the corticosteroids dexamethasone and betamethasone showed the least promising outcomes, with 5/10 (50%) of studies reporting either no improvement or deleterious effects. Indeed, in human studies corticosteroids have been associated with exacerbation of perinatal brain injury, including increased risk of both intraventricular haemorrhage, cerebral palsy and hyperactivity in childhood [8, 106]. The potential for corticosteroids to cause deleterious effects in the perinatal brain are postulated to relate to the stage of neurodevelopment at the time of exposure, the dose and duration of exposure relative to the timing of the insult [107], and their potential to cause hyperglycaemia, which animal and human studies have shown to augment encephalopathy after HI [44, 108]. Furthermore, meta-analysis suggests that prophylactic antibiotics given to women at risk of preterm labour with ruptured membranes are associated with an increased risk of neonatal death and disability [109]. These observations are supported by animal studies, for example treating pregnant rabbits with antibiotics 24 h after intrauterine *E. coli* administration was associated with improved survival but increased white matter cell death [110]. The mechanisms for this are unclear, however it is possible that bacterial lysis promotes the release of bacterial fragments that augment inflammation-induced injury.

Consistent with this hypothesis, we identified two studies in this review that showed increased injury with stand-alone antibiotic or anti-fungal treatments [57, 61]. By contrast, combining antibiotics with the matrix metalloproteinase-9 inhibitor trocade was associated with improved outcomes, suggesting that in cases of fetal or neonatal infection combining antibiotics with an anti-inflammatory intervention could be a more effective approach [57]. Conversely, another study showed that combining antibiotics with the phosphodiesterase inhibitor pentoxifylline did not augment vancomycin-induced protection against Gram-positive bacterial infection, indicating that targeting the right anti-inflammatory mechanism/s to augment antimicrobial treatment is an important consideration [58]. In this analysis anti-cytokine therapies, particularly those targeting the primary effector cytokine IL-1, were most associated with improved outcomes in models of both infection related inflammation and non-infection related inflammation. This raises the possibility that use of anti-cytokine therapies alone or as an adjuvant to antibiotic therapy could be an effective approach to prevent or mitigate inflammation-induced injury in the perinatal brain.

### Who are we treating and when are we treating them?

A key translational consideration for testing potential neuroprotectants is who and when to treat. A minority of therapeutics (8/20; 40%) identified in this review were tested across multiple preclinical models of infection related, non-infection related or combined inflammation. Almost half of the studies (29/59; 46%) started the intervention before or immediately after the insult (within 60 min). Whilst this approach provides useful insight into the early pathophysiology of injury, it unlikely to be practical for clinical translation. Clinically, it is difficult to identify fetuses who are at risk of injury since the positive predictive value of fetal heart rate monitoring and biophysical profiling for predicting adverse neurodevelopmental outcomes is low [111, 112]. Similarly, early neonatal cranial ultrasound is not reliable at detecting ongoing diffuse white matter injury. Instead, its validity has been shown in the setting of advanced severe cystic white matter injury, which is now less common than diffuse non-cystic injury [113–115]. Diffusion magnetic resonance imaging (MRI) has been shown both in preclinical models and in preterm infants to accurately detect acute white matter injury [116–121]. However, it is not feasible to systematically screen all high-risk infants with diffusion MRI in the first few days after birth. Twenty-one out of 59 studies (35%) started the intervention between 1 and 6 h, and 9 studies (15%) started treatment between 18 h and 3 days after the insult. Ideally, pharmacological interventions need to be administered around the time of bulk cell death/injury, which primarily occurs within hours to days after the insult. As well, there is evidence that chronic inflammation makes a contribution to the sub-acute and chronic phases of injury, which develop several days to weeks after the initial insult [9, 122–124]. This suggests that delayed use of immunomodulatory interventions, alone or in combination with interventions that target other pathways of cell damage or repair (e.g. antioxidants, trophic factors, stem cells or stem cell secretomes), could be an effective strategy to mitigate delayed or tertiary brain injury. Ultimately, this raises the need to identify biomarkers of evolving brain injury to facilitate early treatment [125–127], along with understanding the therapeutic window of opportunity for potential interventions in carefully designed animal trials to progress promising therapies from the animal lab to the bedside.

### Assessment of long-term functional and histological outcomes

Another important limitation of the studies identified in this review is that most studies (61%) used survival times of ≤ 7 days, and less than half (22/59 studies; 37%) assessed functional outcomes. Indeed 40% of studies used survival times of hours to 2 days after the insult. Short survival times provide important information about acute histological and functional outcomes, but it is well established that injury evolves many days–weeks after the insult [9, 123, 128] and that functional and histological outcomes are sometimes discordant [96]. Twenty-one out of 59 studies (35%) assessed outcomes beyond 1 week, most (18/21 studies; 85%) reported neuroprotection, however none reported measuring core temperature during treatment or beyond the initial insult. Thus, assessment of histological and functional outcomes in studies beyond the first few hours to days after the insult is an important consideration for future animal trials designed to evaluate the efficacy of potential therapeutics.

### Controlling for potential effects of subject sex on neurological outcomes

Most studies (40/59) reported using subjects of both sexes in the experimental design, however 11 of these studies did not report numbers or ratios of males and females. The remaining studies either did not report the sex of the subjects or tested interventions in males only. Studies investigating the impact of infectious and non-infectious insults have reported sexual dimorphisms in the severity and evolution of immune responses [129], perinatal brain injury [130, 131] and responses to treatment [132, 133]. Four of the 59 studies only used male subjects in their experimental protocol. In addition, only eight studies accounted for sex in outcome reporting. Of these, two stated that a post hoc analysis was performed to assess sex differences between the groups [46]. The remaining five studies reported sex differences as primary outcomes [37, 62, 65], and showed a bias towards neuroprotective effects in males. It remains unclear whether similar differences exist in human trials [11, 134, 135]. Overall, these data raise the need for greater emphasis on evaluating the impact of sex in future animal studies.

### Risk of bias

To evaluate study bias, we used the SYRCLE risk of bias assessment tool. No studies reported random housing of animals. This is a particularly important consideration for small animal (rodent) studies. For example, there is compelling evidence that differences in light exposure, which may vary with respect to rack location, can affect reproduction and behaviour [136, 137]. Additionally, ambient temperature can vary with respect to position of the cage with ambient temperature in the top cage being up to 5℃ higher than the bottom cage [138]. Seventeen out of 40 papers (42%) did not report blinding of examiners during outcome assessments, and 17/40 papers (42%) did not state whether incomplete outcome data were addressed. Only 9/40 papers (23%) reported baseline characteristics, raising the possibility that potential confounders (e.g. unequal distributions of sex, body weight, relevant physiological parameters) may not have been addressed in the analysis. Collectively these data highlight possible inconsistencies in the quality of the data surveyed. We cannot definitively conclude that the methodological issues identified in our analysis affected the outcomes of the studies. Nevertheless, if this critical information is not reported or accounted for in publications, it is difficult to assess the significance of past and future studies in a meaningful way.

## Conclusion

There is an important unmet need to identify and develop effective immunomodulatory interventions for the prevention of perinatal brain injury. Despite many successful preclinical trials, there are no immunomodulatory treatments for perinatal neuroprotection in clinical practice. In this systematic review, we examined preclinical publications between 2012 and 2023 and highlight opportunities to improve the way that preclinical animal trials are designed, carried out and reported to help overcome the ‘translational block’ and close the gap between animal studies and human trials for perinatal neuroprotection. Future studies should evaluate potential therapies in diverse preclinical models that replicate relevant disease pathophysiology, control for iatrogenic changes in temperature that may occur as part of the experimental insult or treatment, address pragmatic treatment regimens that are conducive to clinical application, control for potential effects of subject sex on outcomes, assess long-term functional and histological outcomes, and follow relevant guidelines that mitigate study bias.

### Supplementary Information

**Additional file 1. **Supplementary table 1.

**Additional file 2. **Supplementary table 2.

## Data Availability

The datasets used during the current study are available from the corresponding author upon reasonable request.
